# Supplementary material for: Real-World Outcomes of Patients with Advanced Epidermal Growth Factor Receptor-Mutated Non-Small Cell Lung Cancer in Canada Using Data Extracted by Large Language Model-Based Artificial Intelligence
Source: Curr Oncol. 2024 Apr 2;31(4):1947–60. doi: 10.3390/curroncol31040146 (PMC11049467; doi:10.3390/curroncol31040146)
Supplement: Supplementary file 1 [file curroncol-31-00146-s001.zip › curroncol-2850058-supplementary.pdf]

Supplementary Material

Table S1. 1L treatments in advanced stage NSCLC patients stratified by mutation status at diagnosis

	**Common sensitizing *EGFR* (N=136)**	**Exon 20 insertion (N=8)**	***EGFR* WT (N=338)**
**Any Treatment**	**129 (94.9%)**	**7 (87.5%)**	**232 (68.6%)**
***EGFR* TKI**	**124 (91.2%)**	**1 (12.5%)**	**9 (2.7%)**
Gefitinib	67 (49.3%)	0	3 (0.9%)
Osimertinib	45 (33.1%)	0	1 (0.3%)
Afatinib	10 (7.4%)	1 (12.5%)	5 (1.5%)
Erlotinib	2 (1.5%)	0	0
**ALK TKI**	**0**	**0**	**35 (10.4%)**
Alectinib	0	0	22 (6.5%)
Crizotinib	0	0	13 (3.8%)
**Chemotherapy**	**5 (3.7%)**	**2 (25.0%)**	**127 (37.6%)**
Etoposide	1 (0.7%)	0	6 (1.8%)
Gemcitabine	0	0	5 (1.5%)
Irinotecan	0	0	1 (0.3%)
Platinum	5 (3.7%)	2 (25.0%)	118 (34.9%)
Docetaxel	0	0	1 (0.3%)
Vinorelbine	1 (0.7%)	0	2 (0.6%)
Paclitaxel	0	1 (12.5%)	7 (2.1%)
Pemetrexed	3 (2.2%)	1 (12.5%)	107 (31.7%)
**Immunotherapy**	0	1 (12.5%)	100 (29.6%)
Durvalumab	0	0	6 (1.8%)
Tremelimumab	0	0	6 (1.8%)
Pembrolizumab	0	1 (12.5%)	94 (27.8%)
**Other**	**2 (1.5%)**	**3 (37.5%)**	**15 (4.4%)**
Trastuzumab	0	0	2 (0.6%)
Pertuzumab	0	0	2 (0.6%)
Binimetinib	0	0	6 (1.8%)
Capmatinib	1 (0.7%)	0	0
Entrectinib	0	0	2 (0.6%)
Mg1Magea3	0	0	1 (0.3%)
Lenvatinib	0	0	1 (0.3%)
Selpercatinib	0	0	1 (0.3%)
Savolitinib	1 (0.7%)	0	0
Poziotinib	0	3 (37.5%)	0
Sotorasib	0	0	2 (0.6%)
**No treatment**	**7 (5.1%)**	**1 (12.5%)**	**106 (31.4%)**

	Common sensitizing *EGFR* (N=136)	Exon 20 insertion (N=8)	*EGFR* WT (N=338)
**Time from diagnosis to 1st line treatment initiation (months)**			
Mean (SD)	1.840 (4.778)	3.912 (4.520)	2.461 (4.493)
Median (Range)	0.822 (0.000, 35.769)	2.499 (0.000, 13.644)	1.512 (0.000, 44.054)
Missing	7	1	106

Patients could have received multiple treatments in 1L and therefore percentages may not add up to 100%.
ALK: Anaplastic lymphoma kinase; *EGFR: Epidermal growth factor receptor;* NSCLC: Non-small cell lung cancer; TKI: Tyrosine kinase inhibitor; WT: Wild-type.

Table S2. Evaluation of DARWEN^{TM} AI

Feature	Possible values	Precision	Recall	F1	Overall accuracy
**Histology**	Adenocarcinoma, Non-small cell, Large cell, Adenosquamous, Sarcomatoid	**0.98**	**0.94**	**0.96**	**0.96**
**Smoking status**	Smoker, Former smoker, Never smoker	**0.92**	**0.90**	**0.91**	**0.90**
**Cigarette pack-years**	Numeric				**0.84**
**ECOG at diagnosis**	0–5	**0.97**	**0.97**	**0.97**	**0.97**
**Tumour stage at diagnosis**	I–IV	**0.92**	**0.93**	**0.92**	**0.93**
**Organ level metastatic sites at diagnosis**	Bone, Lung, Brain, Liver	**0.96**	**0.96**	**0.96**	**0.99**
**Biomarkers**	EGFR tested, EGFR positive, EGFR negative, EGFR indeterminate	**0.92**	**0.85**	**0.88**	**0.90**

ECOG: Eastern Cooperative Oncology Group; EGFR: Epidermal growth factor receptor; MET: Mesenchymal epithelial transition factor receptor. Precision: positive predictive value; recall: sensitivity, and F1: harmonic mean of precision and recall.

Figure S1. Summary of included patients

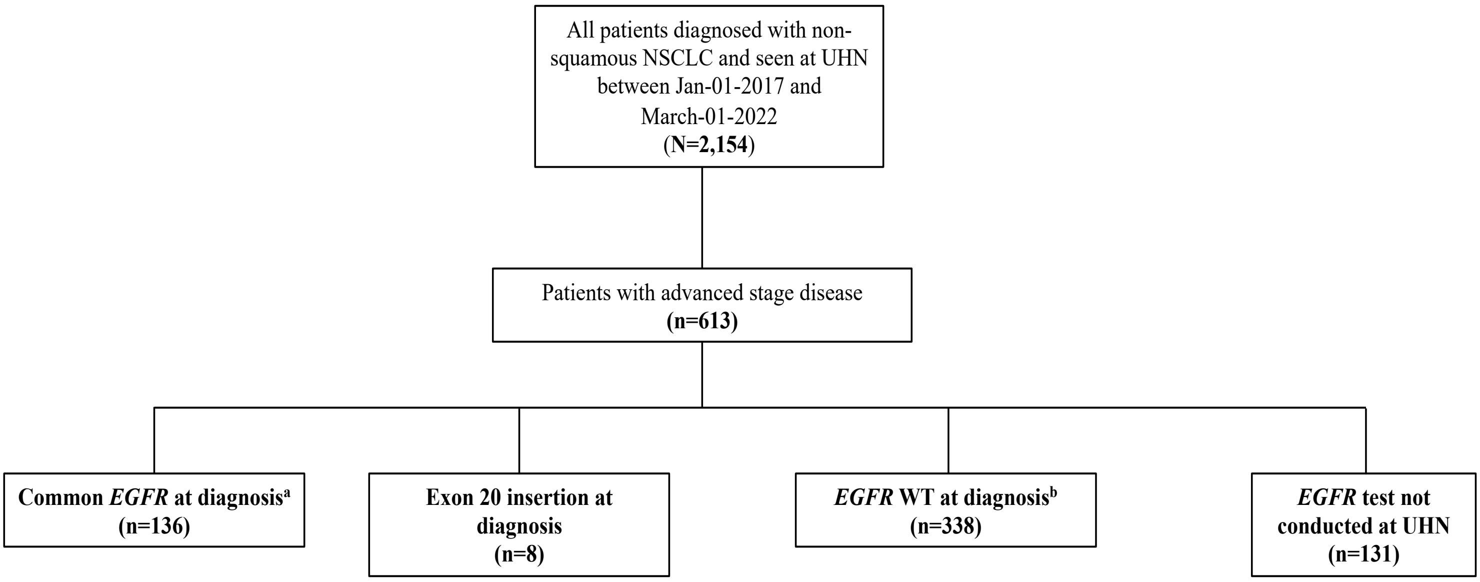

[a]Includes patients with exon 19 deletions or exon 21 L858R mutations; [b]Includes patients with a negative *EGFR* test within 3 months of NSCLC diagnosis but does not exclude the possibility of other mutations; *EGFR: Epidermal growth factor receptor;* NSCLC: Non-small cell lung cancer; UHN-PMCC: University Health Network Princess Margaret Cancer Centre; WT: Wild-type.
